# Postfire reproduction of a serotinous conifer, the giant sequoia, in the Nelder Grove, California

**DOI:** 10.1002/ece3.11213

**Published:** 2024-04-03

**Authors:** Chad T. Hanson, Tonja Y. Chi, Bryant C. Baker, Maya Khosla, Michael K. Dorsey

**Affiliations:** ^1^ Earth Island Institute Berkeley California USA; ^2^ Wildlife Ecologist South Lake Tahoe California USA; ^3^ Wildland Mapping Institute Ventura California USA; ^4^ Wildlife Ecologist Rohnert Park California USA; ^5^ Rob and Melani Walton Sustainability Solutions Service Arizona State University Tempe Arizona USA

**Keywords:** forests, giant sequoia, high severity, Sierra Nevada, wildfire

## Abstract

The giant sequoia, a serotinous conifer naturally occurring in mixed‐conifer forests of the southern and central Sierra Nevada, California, USA, is the world's largest tree species. Giant sequoia reproduction has been severely lacking over the past century, due to fire exclusion, creating a significant conservation threat. Previous research on postfire sequoia reproduction in high‐severity fire areas, relative to low‐ and moderate‐severity areas, is limited. At 6 years postfire, we investigated giant sequoia reproduction in a high‐severity fire area, and nearby low‐/mixed‐severity fire areas, in the Nelder Grove, which burned in 2017 in the Railroad fire. Postfire giant sequoia reproduction was positively correlated with fire severity in terms of density, height (growth), and proportion (relative to other conifer species), and sequoia seedling/sapling density was positively correlated with percent shrub cover. There was no correlation between distance to live sequoia seed source and density of sequoia reproduction. More research is needed in other mixed‐severity fire areas, with larger high‐severity fire patches, to determine whether a similar postfire response occurs elsewhere.

## Graphical abstract

We conducted a rare field investigation of natural postfire giant sequoia reproduction in a large high‐severity fire patch, hundreds of meters from live sequoias, and adjacent lower‐severity areas. Sequoia reproduction density, height growth, and proportion (dominance relative to other conifers) were positively correlated with fire severity, and sequoia seedling/sapling density was also positively correlated with percent shrub cover. There was no correlation with proximity to live sequoias.

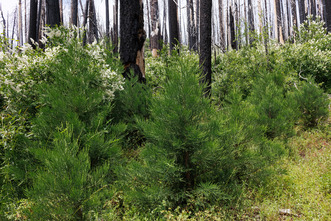

## 1 INTRODUCTION

Globally among conifers, the association with high‐severity fire for effective reproduction, known as serotiny, originated approximately 120–65 million years ago for modern conifer species and 350 million years ago for ancestor species (He et al., 2016; Lamont et al., 2019, 2020). Traits associated with serotiny evolved in response to strong evolutionary selection from high‐severity crown fires. In the western United States, the conservation importance of maintaining or reintroducing high‐severity fire has been recognized for numerous serotinous conifers, such as Rocky Mountain lodgepole pine (*Pinus contorta* var. *latifolia* Engelm., Schoennagel et al., 2003), jack pine (*Pinus banksiana* Lamb., Briand et al., 2015; Pelletier & de Lafontaine, 2023), and the giant sequoia (*Sequoiadendron giganteum* [Lindl.] J.Buchh., Harvey et al., 1980).

The giant sequoia is the most massive tree species on the planet, and also one of the rarest, listed as an endangered species by the International Union for Conservation of Nature (IUCN). Only ~70 naturally occurring giant sequoia groves exist, depending on how grove boundaries are delineated, and these groves span a total cumulative area of ~12,000 hectares, on the western slope of the southern and central Sierra Nevada, California, USA, nearly all of which occurs on lands in U.S. national forests and U.S. national parks (Shive et al., 2022).

The conservation of giant sequoias is closely linked to wildland fire. It has long been recognized that the reproduction of the giant sequoia benefits from wildfires. Sequoias require intense heat from fire to facilitate release of seeds from cones and transform the thick duff and litter on the forest floor into a nutrient‐rich bed of mineral ash, factors which enable seedlings to reach soil and establish roots, while mortality of the tree canopy increases sunlight that aids sequoia seedling growth (Harvey et al., 1980; Stephenson, 1994). A major conservation threat to the giant sequoia has been a “massive failure of sequoia reproduction in groves protected from fire” over the past century due to U.S. federal fire suppression and forest policies that have emphasized short‐term outcomes versus long‐term goals (Stephens et al., 2016; Stephenson, 1994).

Existing published peer‐reviewed research on giant sequoia reproduction in high‐severity fire areas compared with low‐ and moderate‐severity areas is very limited. One study investigated differences in sequoia seedling density as related to fire severity but did not compare reproduction in high‐severity fire areas to low‐/moderate‐severity areas in the same wildfire (Meyer & Safford, 2011). Mutch (1994) presented giant sequoia seedling data for different fire severity categories, providing only qualitative descriptions and did not quantify sequoia seedling densities. Prior research has not examined or addressed how giant sequoias regenerate after higher‐severity fire in areas at large distances (e.g., hundreds of meters) from live sequoia seed trees. Demetry (1995) provided data on giant sequoia seedling density in small high‐severity fire patches 0.1–1.2 ha in size and quantified shrub cover (which was highest in the larger gap sizes). Demetry (1995), however, did not explicitly analyze distance to the nearest live sequoia seed source (using gap edge instead) or explicitly analyze the correlation between percent shrub cover and sequoia seedling density. Demetry (1995) reported higher sequoia seedling density in the larger gap areas (mean gap size = 0.7 ha) that were analyzed, but data were not presented for distances >39 m from gap edges. Meyer and Safford (2011) presented mixed results regarding gap size and giant sequoia reproduction but, like Demetry (1995), did not assess larger gaps (patches) or distances from live sequoia seed sources (e.g., hundreds of meters). Hanson et al. (2024) found no relationship between distance to the nearest live giant sequoia and sequoia seedling density at 2 years postfire in a large high‐severity fire patch in the Redwood Mountain Grove and found that sequoia seedlings were taller and more dominant in the high‐severity area compared with adjacent low‐/moderate‐severity areas. However, these issues have not been investigated in other larger high‐severity fire patches or in other giant sequoia groves.

U.S. land management agencies hypothesize that high‐severity fire patches—especially larger ones—represent a significant conservation threat to giant sequoias, given the potential for such patches to kill mature sequoias and the unknown capacity for this species to reproduce within high‐severity fire patches where live tree seed sources are far away and shrub cover is high (USDA, 2012). The U.S. Forest Service, which oversees the management of approximately half of all extant giant sequoia groves (USDA, 2012), currently emphasizes continued wildfire suppression, lower‐severity prescribed fires, mechanical thinning, and postfire logging, conducted for the stated purpose of reducing fuels and preventing high‐severity wildfire, along with “reforestation” (USDA, 2012, 2022). Following the KNP Complex fire of 2021, Sequoia and Kings Canyon National Parks mapped 177 ha of high‐severity fire in the southern portion of the Redwood Mountain Grove, within a designated Wilderness Area, that was >100 m from the nearest live sequoia seed sources, and suggested a need for “active reforestation” on this basis (USDOI, 2021, 2023).

Despite evidence indicating poor giant sequoia reproduction in lower‐severity fire areas and logged forests (Meyer & Safford, 2011; Stephens et al., 1999), and the well‐documented conservation threat to giant sequoia persistence from fire suppression (Stephenson, 1994), the management approaches described above are being implemented in the face of uncertainty regarding the relationship between giant sequoias and high‐severity fire areas.

**FIGURE 1:**
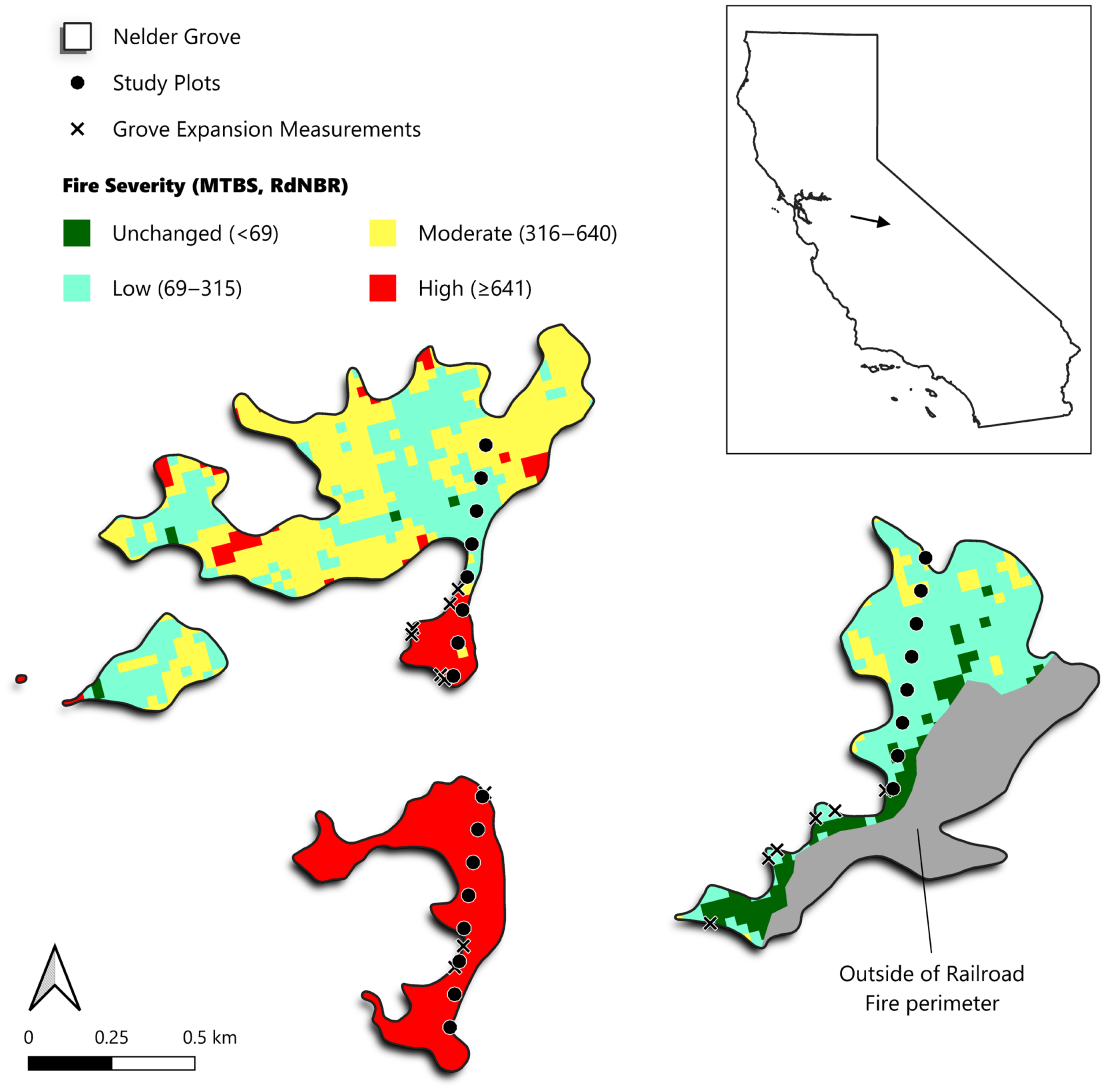
The Nelder Grove study area with fire severity and transect/plot locations.

We assessed giant sequoia reproduction in a high‐severity fire area, and nearby low‐/mixed‐severity fire areas (Figure 1), within the Railroad fire of 2017 in the Nelder Grove of giant sequoias in Sierra National Forest, south of Yosemite National Park. Specifically, we investigated the null hypotheses that (1) density of postfire giant sequoia reproduction would not be correlated with fire severity; (2) height of postfire sequoia seedlings/saplings would not be correlated with fire severity; (3) proportion of postfire sequoia reproduction (sequoia postfire reproduction/ha divided by all postfire conifer reproduction/ha) would not be correlated with fire severity; (4) density (seedlings/saplings per ha) of postfire giant sequoia reproduction would not be correlated with distance to live, cone‐bearing sequoias; and (5) density of postfire giant sequoia reproduction would not be correlated with percent shrub cover.

## 2 METHODS

We surveyed the portions of the Nelder Grove through which the Railroad fire swept in 2017. The Railroad fire burned from August 29 to October 24, 2017, ultimately covering an area of 5021 ha. The fire spread through 111 ha (83%) of the Nelder Grove with mixed fire severity: 46.3% unchanged/low severity, 32.3% moderate severity, and 21.4% high severity (Figure 1).

This area, ranging from 1495 to 1575 m in elevation, is comprised of mixed‐conifer forests dominated by ponderosa pine (*Pinus ponderosa* Douglas ex C. Lawson), sugar pine (*P. lambertiana* Douglas), white fir (*Abies concolor* [Gordon] Lindley ex Hildebrand), incense‐cedar (*Calocedrus decurrens* [Torr.] Florin), giant sequoia in certain locations, and California black oak (*Quercus kelloggii* Newberry), with shrubs mainly consisting of mountain whitethorn (*Ceanothus cordulatus* Kellogg), deer brush (*C. integerrimus* Hook. & Arn.), greenleaf manzanita (*Arctostaphylos patula* Greene), mountain dogwood (*Cornus nuttallii* Audubon ex Torr. & A. Gray), and beaked hazelnut (*Corylus cornuta* Marshall). The fire‐affected areas in the Nelder Grove were not planted with sequoia or other tree seedlings following the fire, so all conifer reproduction was natural. There was 100% mortality of giant sequoias (and other conifer species) in nearly all portions of the high‐severity fire patches; therefore, most of the high‐severity fire areas were ~200–700 m from the nearest live, surviving sequoia after the Railroad fire, creating a unique research opportunity.

We gathered data in three 700‐m transects, each comprised of eight plots spaced by 100 m, covering a wide range of fire severities (Figure 1). Transects were located a priori where a 700‐m linear distance would fit entirely within the Nelder Grove boundaries, which were determined using GIS data provided by the U.S. Forest Service. Plots affected by postfire logging were excluded.

In late June of 2023, at nearly 6 years postfire, we recorded the following within a 5‐m radius of each plot center: (a) the number of sequoia and nonsequoia conifer postfire reproduction (seedlings and saplings); (b) the height (m) of the tallest single sequoia seedling/sapling; (c) percent shrub cover, visually estimated, in the following categories: 0%, 1%–25%, 26%–50%, 51%–75%, and >75% (using the mid‐point of each category for the analysis); and (d) distance (m) to the nearest live, cone‐bearing sequoia, measured with a laser hypsometer for live sequoias within visible distance, but otherwise measured as the distance from the plot center to the nearest low‐/moderate‐severity edge of the sequoia grove. We gathered data in 23 plots. One plot had been affected by postfire logging and was excluded.

For fire severity, we used RdNBR values (Miller & Thode, 2007), based on 1‐year postfire satellite imagery (Figure 1) from the Monitoring Trends in Burn Severity database (www.mtbs.gov).

We also recorded the number of dead sequoia seedlings/saplings >30 cm tall that still retained their foliage within a 5‐m radius of plot centers in moderate‐/high‐severity fire plots to assess the survival rate of sequoia reproduction at 4–6 years postfire (we did not attempt to include seedlings smaller than 30 cm tall given the difficulty of visually finding such seedlings months or longer after they died). We made the conservative assumption that dead sequoia seedlings/saplings that still retained foliage had died within approximately 0–2 years of our summer 2023 field surveys, based on Hart et al. (2015) and Hart and Preston (2020).

For our analyses of all five hypotheses, we used a nonparametric Spearman's rank correlation test (Glantz, 2005; Rosner, 2000).

## 3 RESULTS

**FIGURE 2:**
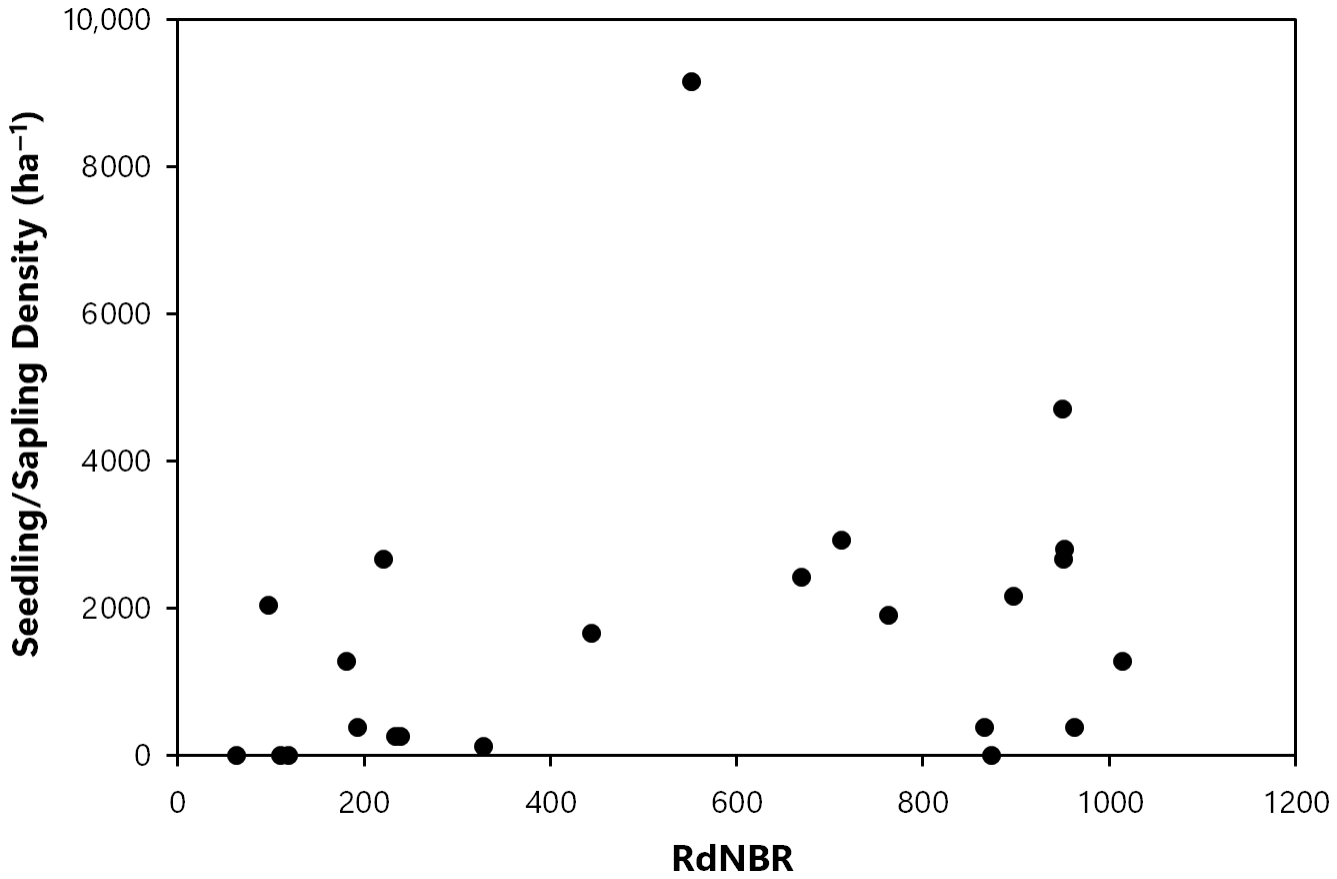
Fire severity (RdNBR values) and giant sequoia seedling/sapling density (stems/ha).

**FIGURE 3:**
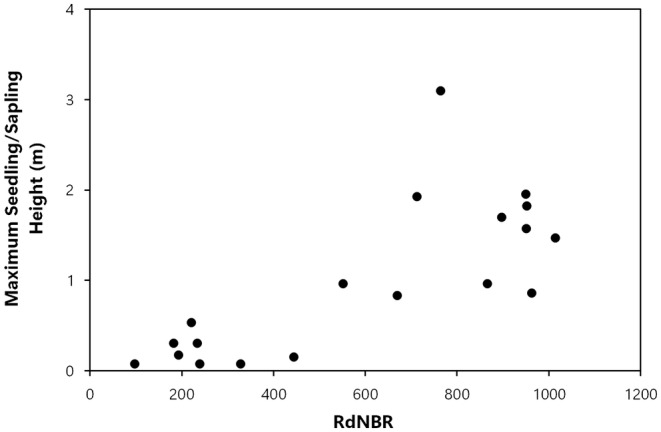
Fire severity (RdNBR values) and maximum giant sequoia seedling/sapling height (m).

**FIGURE 4:**
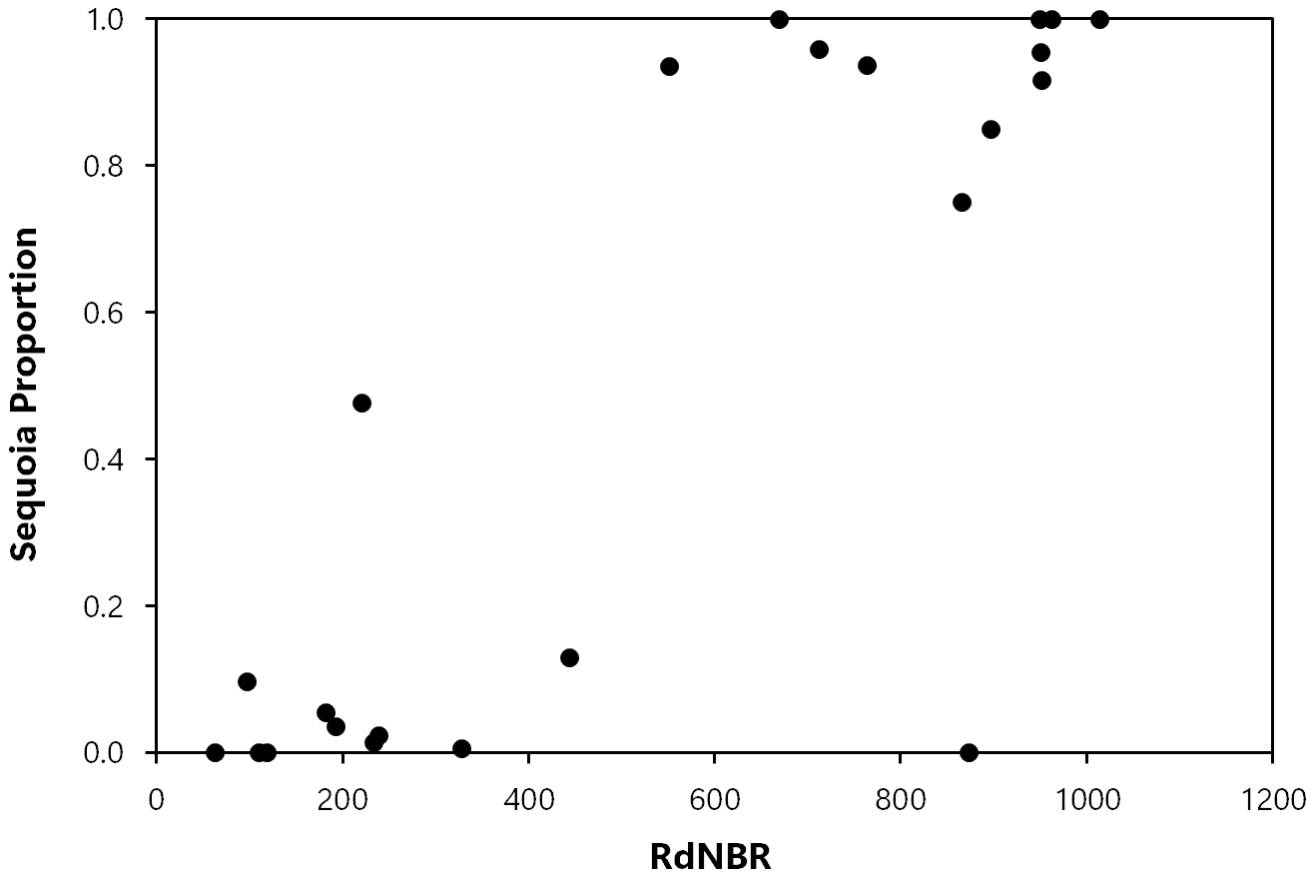
Fire severity (RdNBR values) and giant sequoia regeneration proportion (sequoia postfire reproduction/ha divided by all postfire conifer reproduction/ha).

**FIGURE 5:**
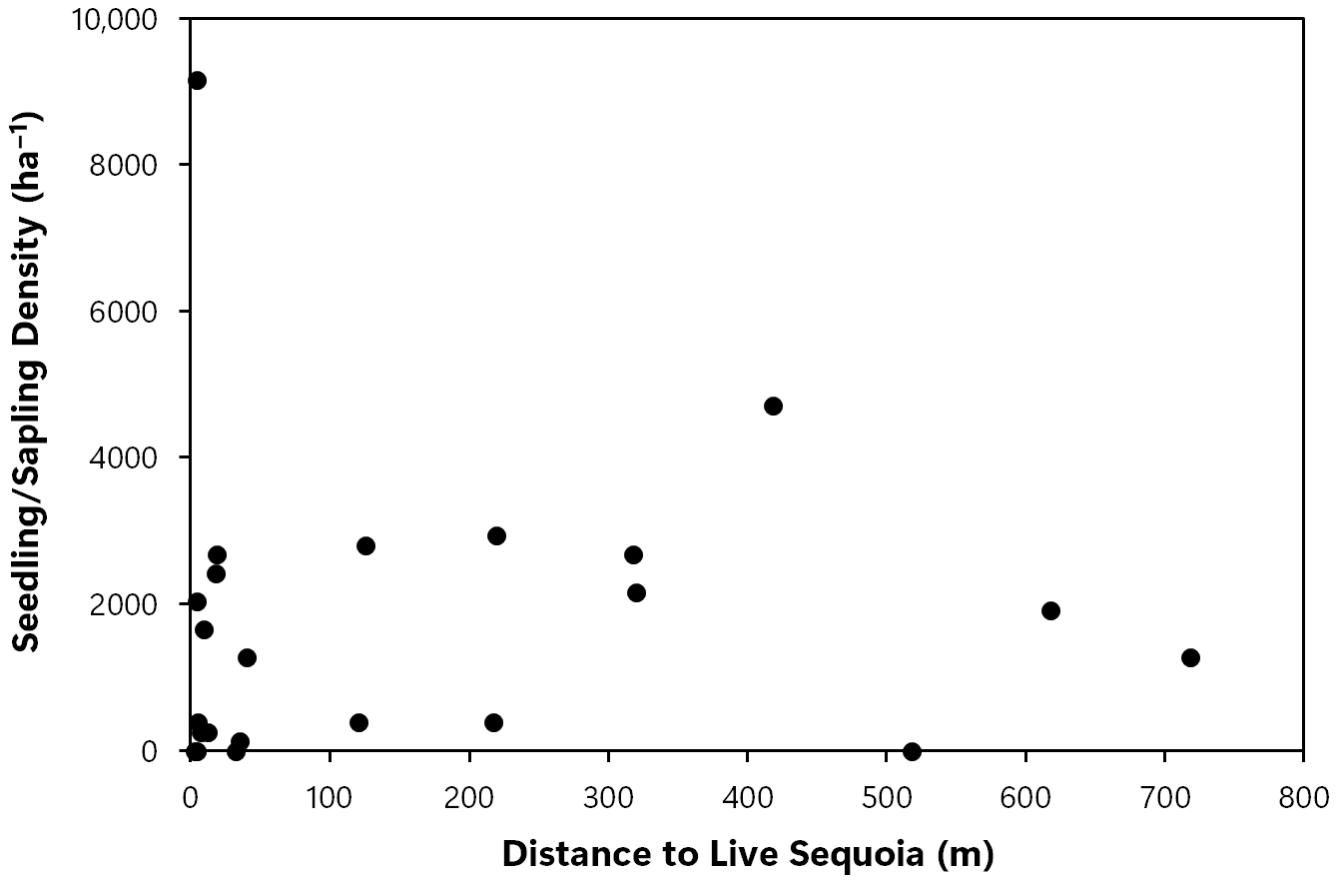
Distance (m) to the nearest live sequoia seed source and giant sequoia seedling/sapling density (stems/ha).

**FIGURE 6:**
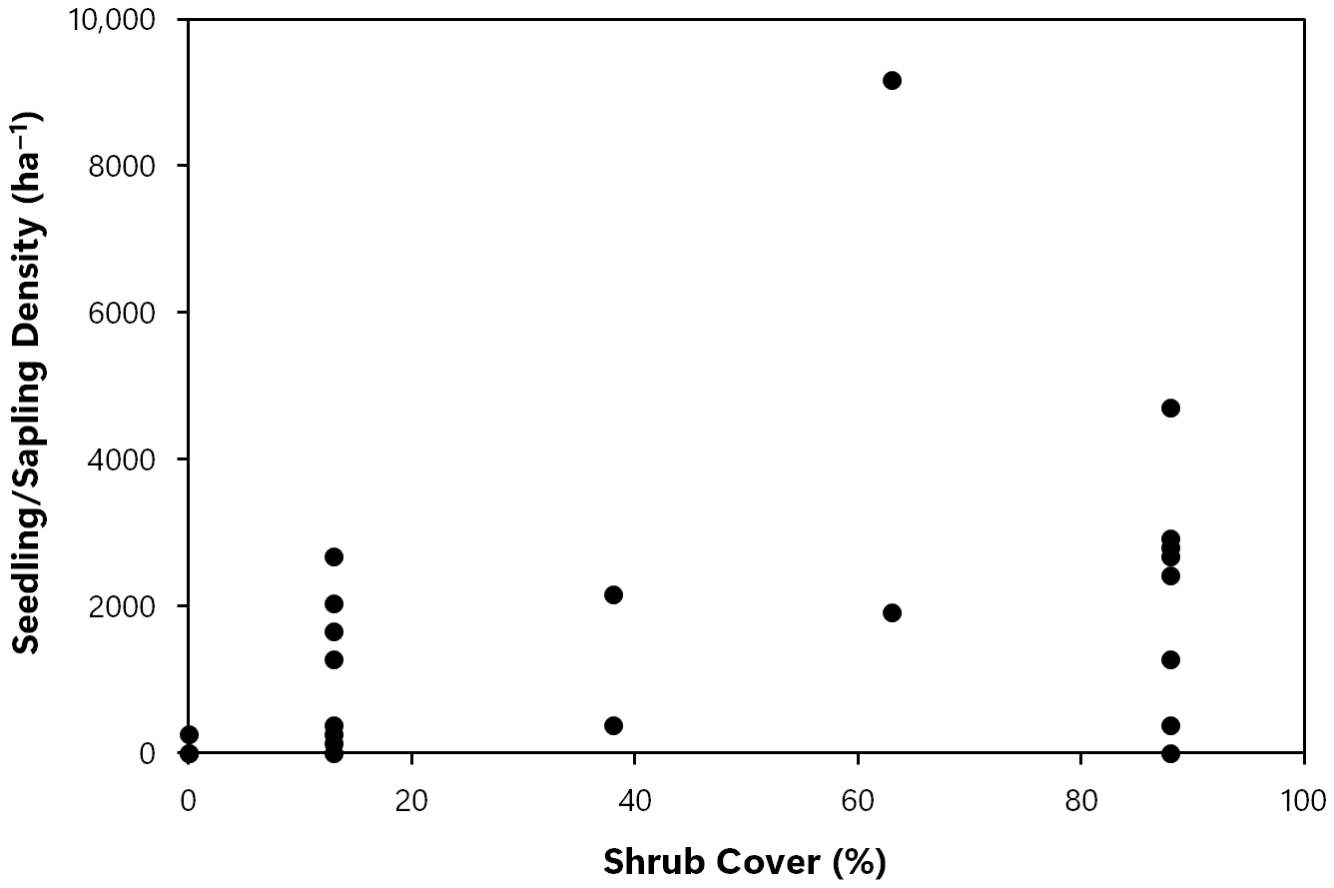
Percent shrub cover and giant sequoia seedling/sapling density (stems/ha).

The density of postfire sequoia reproduction was positively correlated with increasing wildfire severity (*r*_s_ = .425, *p* = .043, Figure 2), and the null hypothesis was rejected. The maximum height of giant sequoia seedlings/saplings (*r*_s_ = .727, *p* < .001, Figure 3), and sequoia regeneration proportion (sequoia postfire reproduction/ha divided by all postfire conifer reproduction/ha) (*r*_s_ = 0.728, *p* < .001, Figure 4), were also positively correlated with increasing wildfire severity, and the null hypotheses were rejected. The null hypothesis was not rejected with regard to the correlation between postfire sequoia reproduction density and distance to the nearest live, cone‐bearing sequoia seed source, as there was no correlation between these two variables (*r*_s_ = .241, *p* = .267, Figure 5). There was a significant positive correlation between percent shrub cover and density of postfire sequoia reproduction (*r*_s_ = .557, *p* = .006, Figure 6), and the null hypothesis was rejected. In high‐severity fire plots, only 6.6% of sequoia seedlings/saplings >30 cm tall had died over the past 2 years, indicating an annual mortality rate of 3.3%, and an annual survival rate of 96.7%.

## 4 DISCUSSION

**FIGURE 7:**
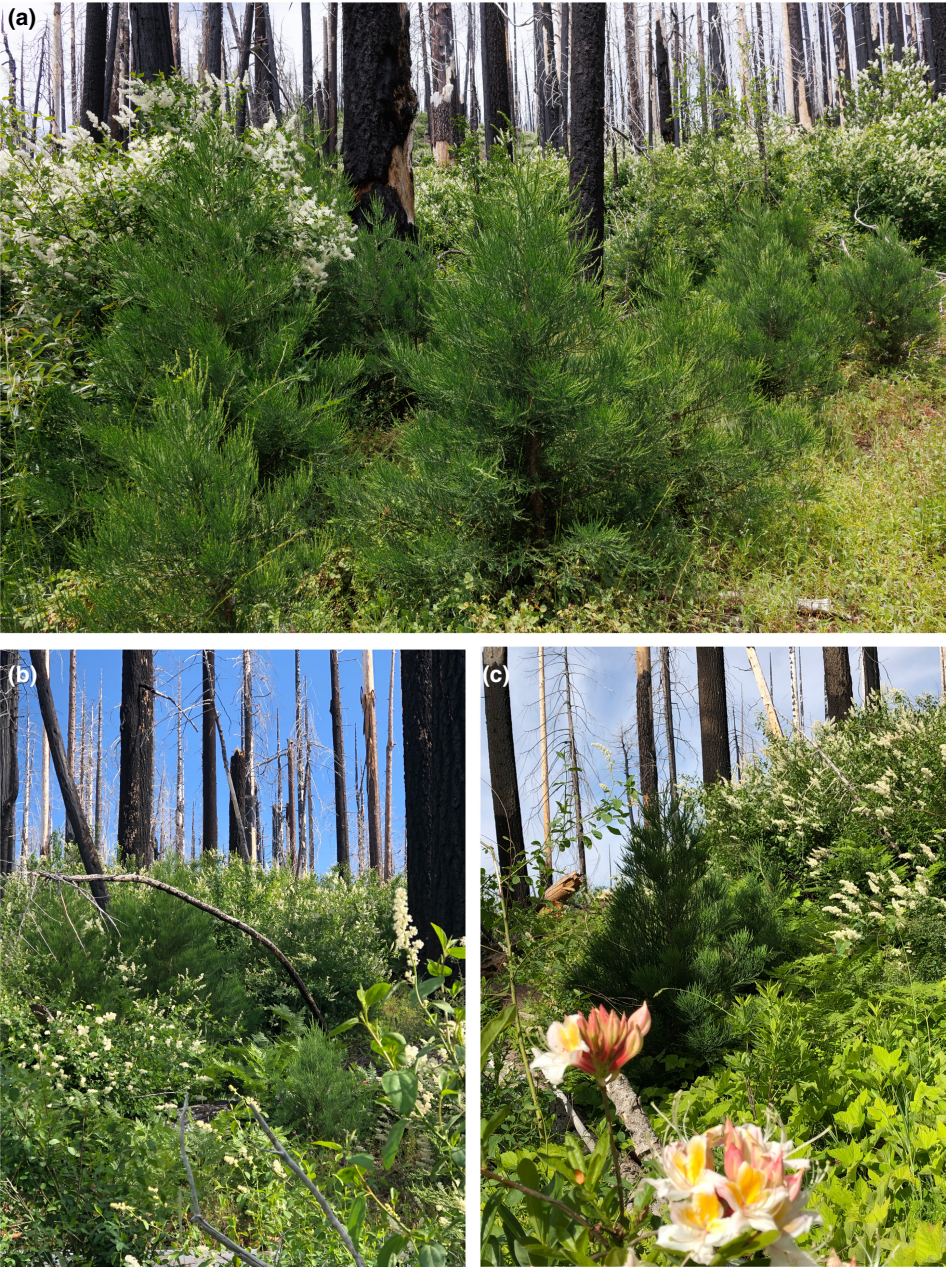
Natural giant sequoia reproduction in a high‐severity fire area hundreds of meters from the nearest live sequoia seed source in the Nelder Grove: (a) in an area of heterogeneous shrub cover; (b) ~8 giant sequoia saplings in a location with nearly 100% shrub cover; and (c) giant sequoia saplings, 2–3 m tall, with montane chaparral and western azaleas.

In the Railroad fire of 2017 within the Nelder grove, we found vigorously growing giant sequoia seedlings and saplings within high‐severity fire areas, and natural postfire succession that is lush in appearance. Giant sequoia reproduction density, growth (height), and proportion (relative to other conifer species) were positively correlated with fire severity. To put our findings in perspective, in higher‐severity areas with ≥50% basal area mortality from the fire (RdNBR ≥477; Miller et al., 2009, Hanson, 2015), median giant sequoia reproduction was 9 times denser, 7 times taller, and 48 times more dominant (relative to other conifers) than it was in areas with <50% basal area mortality (Figures 2, 3, 4, 5, 6, Appendix S1). Distance from live, cone‐bearing sequoias was not a significant factor. Even at large distances from the nearest live sequoia seed source, for example, ~300–700 m, we found >2000 sequoia seedlings/saplings per hectare (Figures 5 and 7). These results are broadly consistent with the findings of Hanson et al. (2024) in the Redwood Mountain Grove at 2 years postfire and also with the reproductive advantages conferred by higher‐severity fire among serotinous species.

We found very high survival of giant sequoia regeneration in the high‐severity fire areas. Some caution is appropriate with regard to this finding, in light of the fact that dense shrub cover in certain areas prevented us from being able to reliably find dead sequoia seedlings <30 cm tall. Additional research is needed on this subject.

Our finding of significantly higher sequoia reproduction in areas with the highest shrub cover is broadly consistent with descriptive data provided in Demetry (1995). It is not known whether this finding represents the fact that both giant sequoias and native shrubs proliferate best in high‐severity fire areas, or if it suggests that giant sequoia reproduction benefits in some ways from shrub cover. More research is needed to determine whether this relationship appears in other large high‐severity fire patches at 5 or 6 years postfire within sequoia groves and to better understand the nature of the relationship. Moreover, a posteriori data that we gathered suggest a need for future study of the potential for higher‐severity fire areas to facilitate sequoia grove expansion (Appendix S2).

**FIGURE 8:**
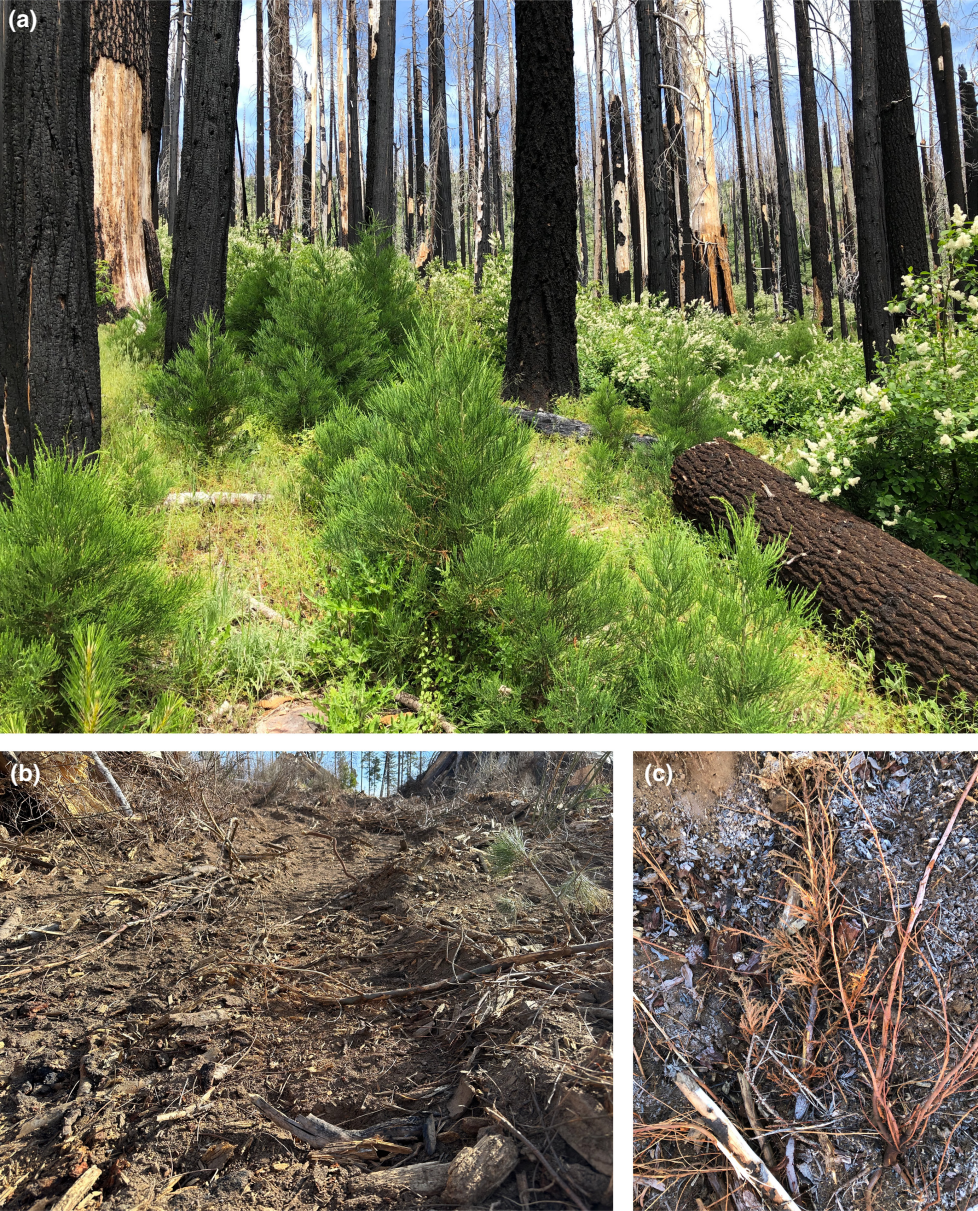
(a) Natural giant sequoia reproduction in a high‐severity fire area in the Nelder Grove, (b) adjacent postfire logging at the edge of the Nelder Grove, where an estimated 83% mortality of giant sequoia seedlings and saplings occurred, based on limited a posteriori data (Appendix S3), and (c) crushing of a giant sequoia sapling by logging machinery.

While acknowledging the historical occurrence of small high‐severity fire patches in giant sequoia groves, Shive et al. (2022) recently recommended an intensive management regime, including mechanical thinning and lower‐severity prescribed fires, designed to prevent larger high‐severity fire patches. A “lower severity” fire regime was assumed by Shive et al. (2022) to promote resilient giant sequoia groves. Shive et al. (2022) did not sufficiently address how the generally low levels of sequoia reproduction in a lower‐severity fire regime would effectively replenish sequoia grove populations and establish ecological resilience over time (Stephenson, 1994), or assess sequoia seedling mortality in areas applying ground‐based machinery for thinning (Donato et al., 2006). Shive et al. (2022) also did not acknowledge the increase in overall tree mortality that would result from intensive management activities (Bartowitz et al., 2022), the failure of tree removal to achieve fire management objectives due to microclimate effects caused by such removal (Lesmeister et al., 2021), or the body of scientific literature indicating that no tree cutting or removal is needed prior to conducting prescribed fires (e.g., Knapp et al., 2005; van Mantgem et al., 2011). Such management, including postfire logging, was recently authorized by the U.S. Forest Service in several giant sequoia groves (USDA, 2022), including the Nelder Grove (Figure 8).

Statements made in a pre‐print study (Stephenson et al., 2023) and (Soderberg et al., 2024) are being used to guide a large U.S. National Park Service project implementing expansive sequoia seedling planting of nursery‐grown stock into high‐severity fire patches within designated Wilderness Areas (USDOI, 2023). Both studies state that natural postfire reproduction in high‐severity areas would not be adequate to restore high‐severity burned sequoia groves, leading to regeneration failure and loss to portions of sequoia groves (Soderberg et al., 2024; Stephenson et al., 2023). Neither of the studies investigated whether there is a postfire sequoia seedling density threshold below which regeneration failure will likely occur, and neither presented data on postfire sequoia reproduction in high‐severity fire areas compared to low‐/moderate‐severity areas. Notably, the median sequoia seedling density in higher‐severity areas in our data (2292/ha) is 14 times higher than the median density of 160/ha at 5 years after prescribed fires in Stephenson et al. (2023), and sequoia seedlings were completely absent in one‐third of plots in Stephenson et al. (2023) at 5 years following prescribed fire. While the *mean* seedling density at 5 years postfire in Stephenson et al. (2023) was high, this value was overwhelmingly driven by four plots with very high densities, out of a total of 36 plots. Reporting means, rather than medians, with such heavily skewed data tends to cause a misleading impression of results (Glantz, 2005; Zar, 2010). Thus, important management decisions, with potentially long‐term effects, are being made in the absence of scientific evidentiary support and essential knowledge.

Soderberg et al. (2024) reported lower postfire giant sequoia seedling density at the upper end of the high‐severity fire spectrum (particularly ≥900 RdNBR) at 1 year postfire. Our results, at 6 years postfire, do not suggest a decrease. In our Nelder Grove data, median sequoia seedling density for areas with RdNBR values <477 was 255/ha, while areas with RdNBR values 477–899 had a median sequoia seedling density of 2165/ha, and areas with RdNBR ≥900 had a median density of 2674/ha (nearly 17 times higher than the median sequoia seedling density at 5 years after prescribed fires in Stephenson et al., 2023). As Soderberg et al. (2024) reported results 1 year postfire, our results reported 6 years postfire, may not be inconsistent with theirs. Sequoia seedling density may be relatively lower in areas with RdNBR values ≥900 at 1 or 2 years postfire, and such areas may provide good conditions for sequoia seedling survival, growth, and perhaps additional germination and establishment, which could shift the relative seedling density higher in such very high‐severity areas by several years postfire. Additional research is needed to further address this.

Coppoletta et al. (2016) reported a somewhat higher probability of high‐severity reburn in high‐severity fire patches with high shrub cover, but the overall high‐severity fire percentage in reburns within earlier high‐severity fire patches was low, only 9.3% (i.e., 90.7% low‐/moderate‐severity fire on average if and when a high‐severity fire patch reburned). Further, shrub cover is ephemeral following high‐severity fire, and shrubs soon die back and recede, within as little as 12 years postfire, as postfire conifer regeneration grows denser and taller and natural succession advances (Hanson, 2018). Therefore, the young, naturally regenerating sequoia forests in high‐severity fire patches are more likely to experience low‐/moderate severity fire if a reburn occurs. Importantly, even young, small giant sequoias (e.g., 10 cm in diameter at breast height) have been reported to have 100% survival even when fire kills up to 90% of their crown foliage, and survival remains high until percent crown foliage mortality exceeds 95% (Stephens & Finney, 2002).

Moreover, giant sequoias grow rapidly after high‐severity fire (Meyer & Safford, 2011) and can produce cones with viable seeds, capable of germination, when they are 10–14 years old (Hartesveldt et al., 1975). Wind gusts capable of blowing giant sequoias seedlings >500 m occur multiple times each year in sequoia groves (Harvey et al., 1980), creating potential for sequoia seedling germination in the interior of larger high‐severity fire patches, even if a high‐severity reburn has occurred. In the mixed‐conifer forest types inhabited by giant sequoias, high‐severity fire naturally includes both small patches and large ones hundreds of hectares in size, and occurs on cycles (rotation intervals) typically spaced by three or four centuries on average (Baker, 2014; Baker & Hanson, 2017). Within this spatiotemporal natural disturbance context, giant sequoias have multiple mechanisms through which they can reach maturity as subsequent fires occur after a stand‐initiating event.

Further research will be needed in other recent fire areas to determine whether findings emerge that are similar to our results for the Nelder grove. Our results suggest the need for some caution and scrutiny regarding current management proposals that assume actions conducted with the goal of preventing high‐severity fire will promote resilience and conservation of giant sequoia groves.

Giant sequoia reproduction after high‐severity fire is heterogeneous, and such heterogeneity appears to be a natural, intrinsic property of giant sequoia groves (Stohlgren, 1993). Based on current data, we recommend that land managers actively monitor the natural succession of giant sequoias in the years following high‐severity fire. In the interim, we believe the existing evidence indicates that intensive forest management interventions to facilitate giant sequoia reproduction are unwarranted and potentially counter‐productive (Figure 8). We recommend an emphasis on wildland fire use, that is, allowing more lightning ignitions to burn without fire suppression in more remote forests (Stephens et al., 2021; Stephens & Finney, 2002) at natural rotation intervals (Baker, 2014, 2017), and embracing the ecological benefits of larger mixed‐severity wildfires that occur, rather than mechanized and artificial forest management and wildfire suppression approaches that may have unintended consequences (Figure 8).

## AUTHOR CONTRIBUTIONS

**Chad T. Hanson:** Conceptualization (lead); data curation (lead); formal analysis (lead); funding acquisition (lead); investigation (lead); methodology (lead); project administration (lead); resources (equal); software (supporting); supervision (lead); validation (equal); visualization (equal); writing – original draft (lead); writing – review and editing (lead). **Tonja Y. Chi:** Conceptualization (supporting); investigation (equal); methodology (equal); validation (equal); visualization (supporting); writing – original draft (supporting); writing – review and editing (equal). **Maya Khosla:** Investigation (equal); methodology (supporting); writing – review and editing (equal). **Bryant C. Baker:** Investigation (equal); methodology (supporting); software (lead); writing – review and editing (equal). **Michael K. Dorsey:** Conceptualization (supporting); investigation (equal); methodology (supporting); validation (supporting); writing – review and editing (equal).

## FUNDING INFORMATION

This research was funded by the Environmental grantmaking foundation (grant no. 2023).

## CONFLICT OF INTEREST STATEMENT

The authors declare no conflicts of interest.

## Supporting information


Appendix S1.

Appendix S2.

Appendix S3.

## Data Availability

The raw data for this study, including plot coordinates, are available in Appendix S1.
