# Supplementary material for: Postfire reproduction of a serotinous conifer, the giant sequoia, in the Nelder Grove, California
Source: Ecol Evol. 2024 Apr 3;14(4):e11213. doi: 10.1002/ece3.11213 (PMC10990047; doi:10.1002/ece3.11213)
Supplement: Supplementary file 2 — Appendix S2. [file ECE3-14-e11213-s002.docx]

# Giant Sequoia Post-Fire Regeneration Outside of Grove Boundaries

In the course of gathering field plot data, whenever we were within 10 m of the Nelder Grove boundaries on our transects, or while hiking from one transect to another, we recorded the maximum distance (m) outside of the grove boundary at which we could find post-fire sequoia reproduction, in 10-m-wide transects extending perpendicular away from the grove boundary, to assess the potential for grove expansion following the 2017 Railroad fire. We report these observations as descriptive data below.

Maximum distance (m) outside of the Nelder Grove boundaries at which post-fire sequoia reproduction was found along high-severity fire (RdNBR >640) grove boundary locations and low/moderate-severity fire (RdNBR <640) grove boundary locations.

| Low/Moderate | High |
| --- | --- |
| 0 | 20 |
| 0 | 28 |
| 0 | 34 |
| 0 | 43 |
| 9 | 51 |
| 12 | 52 |
| 31 | 52 |
| - | 55 |
